# Association of lipocalin-2 level, glycemic status and obesity in type 2 diabetes mellitus

**DOI:** 10.1186/s13104-017-2604-y

**Published:** 2017-07-14

**Authors:** Areej E. Elkhidir, Halima B. Eltaher, Abdelrahim O. Mohamed

**Affiliations:** 1 Department of Biochemistry, Faculty of Medicine, University of Khartoum, P O Box 102, Khartoum, Sudan; 2 Department of Biochemistry, School of Medicine, Ahfad University of Women, Omdurman, Sudan; 3 Al-Neelain Institute for Medical Research, Al-Neelain University, Khartoum, Sudan

**Keywords:** Type 2 diabetes mellitus, Obesity, Lipocalin-2, Waist circumference, Sudan

## Abstract

**Background:**

Management of type 2 diabetes mellitus aims to maintain a normal glycemic status, which if not, it may lead to acute and/or chronic diabetic complications. Earlier studies found Lipocalin-2 elevated in complications associated with type 2 diabetes mellitus such as ischemic heart disease. These lipocalin-2 changes had been linked to obesity and uncontrolled diabetes. So, it could be useful to understand the effect of glycemic control and obesity on lipocalin-2.

**Methods:**

This was a case control study. Fifty-seven patients with type 2 diabetes and 30 non-diabetic controls participated after getting a written consent. Weight (kg), height (m) and waist circumference (cm) were measured then the body mass index (kg/m^2^) was determined. Blood samples were collected after an overnight fasting. HbA1c, lipid profile and serum creatinine were measured using enzymatic methods. Lipocalin-2 was measured using sandwich ELISA.

**Results:**

Lipocalin-2 was found significantly higher in patients with type 2 diabetes (P = 0.001). However, it had no significant correlation with any of the studied variables. Females had elevated BMI compared to males in the patients group (P < 0.001). HbA1c, serum creatinine, LDL and total cholesterol were elevated in patients with diabetes (P < 0.02). HDL was lower in the patients (P = 0.002). Significant elevation in HbA1c was found in male patients (P = 0.028) compared to female patients. Patients were further classified into controlled, uncontrolled diabetics, obese and non-obese. There was a significant elevation in waist circumference in uncontrolled diabetics compared to controlled ones. Lipocalin-2 had no significant changes between controlled and uncontrolled diabetics nor non-obese and obese patients.

**Conclusion:**

Patients with type 2 diabetes mellitus have elevated level of serum lipocalin-2. There was no significant association found between lipocalin-2 and glycemic control nor obesity.

**Electronic supplementary material:**

The online version of this article (doi:10.1186/s13104-017-2604-y) contains supplementary material, which is available to authorized users.

## Background

Type 2 diabetes mellitus has become a worldwide epidemic. The prevalence was estimated to be 6.4% among adults in 2010. The number of people affected will continue rising dramatically, and number of people will double from 285 million to 439 million from 2010 to 2030 [1]. High risk of disabilities and mortality rates associated with diabetes include neuropathy, retinopathy, nephropathy and atherosclerotic vascular disease. Their estimated prevalence in North Africa is 8.1 to 41.5%, 21 to 22%, 6.7 to 46.3% and 21.9 to 60% for retinopathy, albuminuria, nephropathy and neuropathy respectively [2]. It is the fifth leading cause of deaths globally. Type 2 diabetes mellitus is a major health problem in Sudan with prevalence about 2.6% [2]. About 7% of hospital admissions in Sudan are patients with diabetic complications [3].

Although the direct cause of diabetes mellitus is lack of insulin or decreased sensitivity to it [4, 5], in about 80% of patients with DM2, obesity is the major predisposing factor [5]. Increased body weight is considered the sixth most important risk factor contributing to the overall burden of disease worldwide [6]. Prevalence of obesity has increased globally in the past 30 years from 28.8 to 36.9% in men and 29.8 to 38.0% in women [7]. Both DM2 and obesity (mainly abdominal obesity) are predisposing factors for metabolic and vascular complications including ischemic heart diseases, which are the top leading causes of death in the Arab world and a leading cause of disability-adjusted life years (DALYs) in men [5, 8].

Lipocalin-2 (LCN2) is neutrophil gelatinase-associated lipocalin (NGAL). It belongs to lipocalin family which functions as transporters for hydrophobic molecules [9]. LCN2 is firstly isolated in 1989 from mouse kidney cells [9]. Then it was isolated in humans as a protein associated with human neutrophil gelatinase in 1993 [10]. Recent studies defined lipocalin-2 as an adipokine that is secreted mainly from adipose tissue [11]. LCN2 expression and secretion increase after conversion of preadipocytes to mature adipocytes [12]. LCN2 expression is induced by many pro- and anti-inflammatory cytokines and factors such as lipopolysaccharide (LPS), tumor necrosis factor-α (TNF-α), IL-1β, IL-6 or IL-17 in a variety of cell types [13]. LCN2 as an adipokine has bacteriostatic effects within the innate immune response paired with protective functions in chronic inflammation of the airway system. It has a role as a biomarker for renal injury [13]. In regard to diabetic complications such as atherosclerosis, recent evidence suggests that LCN2 plays a pivotal role in vascular remodeling and plaque instability specifically LCN2 expressed in macrophages [13]. LCN2 has an important role in glucose homeostasis and insulin sensitivity [11].

Recent studies have found significant association between LCN2 and diabetic complications. Some studies found significant correlation between LCN2 and atherosclerosis in patients with diabetes mellitus, obesity and the metabolic syndrome [14, 15]. Other studies correlated it with uncontrolled DM2 [16] and diabetic kidney diseases [17, 18].

This study aimed to investigate the level of lipocalin-2, glycated hemoglobin (HbA1c) in Sudanese patients with type 2 diabetes mellitus. As atherosclerosis and diabetic kidney diseases was reported to have correlation with elevated level of LCN2, lipid profile and creatinine were also investigated. Additionally, the study aimed to investigate the correlation between LCN2, other investigations, BMI and waist circumference.

## Methods

It was a case control study, which was conducted at Jabir Abu Elez diabetic center, Khartoum, Sudan. Fifty-seven patients with type 2 diabetes mellitus and 30 non diabetics participated in the study. The sample size was calculated using the equation: ${\text{n}} = {\text{z2pq}}/{\text{d2}}$ (n = sample size; z = the standard normal deviate, usually set at 1.96, which corresponds to the level of the 95% confidence level; p = the proportion to the target population. Age and sex matched individuals were enrolled after obtaining a written consent which was approved by University of Khartoum, Faculty of Medicine ethics committee. Subjects were included according to the following criteria: age more than 44 and less than 60 years with type 2 DM because adults aged 45–64 were the most diagnosed age group and who attended the diabetes clinic on Sundays and Tuesdays during the study period between March and August 2015.

All participants were interviewed. History taking and examination were performed using the data collection sheet; age, sex, duration of the disease and blood pressure. Body mass index (BMI) was calculated as weight/height^2^ (kg/m^2^) categorized as normal weight (<25.0 kg/m^2^), or overweight plus obese (≥25 kg/m^2^) according to the WHO criteria [19]. Waist circumference was measured midway between the lower rib margin and the iliac crest considered normal if the waist circumference of ≥94 cm (~37″) in men and ≥80 cm (~31.5″) in non pregnant women are used as cut offs for central obesity in the European Union.

### Blood sampling

Blood samples were obtained in the morning after 6–8 h overnight fast using EDTA and plain containers and were processed as follows; EDTA blood samples were used for HbA1c analysis during the same day. Then plasma and serum were separated with centrifugation and were immediately stored at −80  °C for subsequent assay.

### Laboratory analyses

HbA1c was measured using boronate-affinity binding enzymatic methods using Nycocard HbA1c (Axis-shield PoC AS, Oslo, Norway) [20]. Diabetic patients were classified into controlled (HbA1c < 7.5%) and uncontrolled (HbA1c ≥ 7.5%) [21]. Lipid profile and serum creatinine were measured using enzymatic methods from Biosystems reagents & instruments, Barcelona, Spain [22].

Lipocalin-2 was measured in plasma by sandwich Elisa using Human Lipocalin-2/NGAL ELISA (BioVendor research and diagnostic products, Czech Republic) [23].

### Statistical analysis

IBM SPSS statistics (V.20.0, IBM Corp., USA, 2010) was used for data analysis. Results were expressed as the mean ± SEM. Comparison between two independent mean groups was done using student’s *t* test. Pearson’s correlation coefficients were used to assess associations between variables. P values reported were two-tailed, and P at 0.05 was considered significant.

### Limitations section

Although this study was carefully prepared, there are unavoidable limitations. First, the sample size was relatively small which might have potential impact on the findings. This was due to time and budget as second limits. It would be better if the sample recruiting time was long enough plus having funding resources instead of personal ones.

## Results

Eighty-seven individuals participated in the study and were divided into two groups 57 diabetic patients and 30 controls. Anthropometric, clinical and biochemical variables of the studied subjects were presented in Tables 1 and 2. Lipocalin-2 and HbA1c were significantly elevated in the patients’ group compared with the controls, P-value <0.001. There was also significant elevation of low density lipoprotein (LDL), total cholesterol and serum creatinine in patients with diabetes compared with controls, P-value <0.02. High density lipoprotein (HDL) was found significantly lower in patients with diabetes P = 0.002. BMI and triglycerides (TAG) differences between the groups were not significant (P-value >0.05). While waist circumference was nearly significantly higher in patients, P = 0.063.

**Table 1:** Clinical and anthropometric characteristics of patients with DM2 and controls (mean ± standard deviation)

*n*	Patients	Controls	P value
	57	30	
Gender (M/F)	26/31	18/12	
Duration (years)	9 ± 6	–	–
Age (years)	51 ± 4	50 ± 4	–
BMI (kg/m^2^)	27.9 ± 5.3	27.4 ± 5.4	0.663
Waist circumference (cm)	96.4 ± 10.7	91.4 ± 11.8	0.063

**Table 2:** Laboratory characteristics of patients with DM2 and controls (mean ± standard deviation)

*n*	Patients	Controls	P value
	57	30	
Lipocalin-2 (ng/μl)	87.68 ± 53.28	45.32 ± 46.15	0.001
HbA1c (%)	9.3 ± 2.3	5.8 ± 0.9	<0.001
Serum creatinine (mg/dl)	1.0 ± 0.3	0.7 ± 0.2	<0.001
LDL (mg/dl)	90.1 ± 32.1	66 ± 28.5	0.001
HDL (mg/dl)	48.3 ± 17.5	60.3 ± 13.9	0.002
Triglyceride (mg/dl)	135.4 ± 52.4	139.6 ± 52.9	0.724
Total cholesterol (mg/dl)	171.1 ± 39.9	151.6 ± 30.2	0.022

Table 3 shows LCN2 level comparison between different groups according to gender, HbA1c and BMI. According to gender distribution, there was a significant difference in LCN2 between male patients compared to male controls in serum (P < 0.05). Equivalently, there was a significant difference in LCN2 between female patients and controls. But no difference was seen between male and females in the patient group (P > 0.05). However, there was insignificant difference in LCN2 between controlled, uncontrolled, non-obese and obese diabetics with P-value >0.05.

**Table 3:** Comparison of lipocalin-2 level between groups according to gender, HbA_1c_ %, BMI (mean ± standard deviation)

	Patients		Controls		P1	P2	P3
	Males	Females	Males	Females			
*N*	26	31	18	12			
Lipocalin-2 (ng/μl)	79.7 ± 51.3	94.4 ± 54.8	45 ± 52.1	45.8 ± 38.4	0.305	0.04	0.01

	Controlled T2DM	Uncontrolled T2DM	P
Lipocalin-2 (ng/μl)	108.4 ± 67.1	85.6 ± 48.4	0.267

	Non obese *N* = 17	Obese *N* = 36	P
Lipocalin-2 (ng/μl)	93.8 ± 56.1	86.2 ± 53.5	0.639

P1 for male and female patients, P2 for the male patients and male controls, P3 for the female patients and female controls

The anthropometric, clinical and biochemical variables in both studied groups according to gender distribution shows (see Additional file 1: Table S1). As we had few missing data in height and weight, the BMI was not determined in four patients. Concerning BMI, it was significantly elevated in females compared to males in patients group at P < 0.001. HbA1c was significantly elevated in males compared to females of patients group at P = 0.028. There was significant difference between male patients compared to male controls in serum creatinine, LDL and HDL (P < 0.05). There was significant difference in LDL and TAG between female patients and controls. Interestingly about 88.9% of our females had waist circumference exceeding 80 cm (according to European standard), some of them were within normal weight.

Patients with type 2 diabetes mellitus were further classified according to HbA1c % into controlled patients with diabetes (HbA1c < 7.5%) and uncontrolled (HbA1c > 7.5%). In addition, patients were also classified according to BMI into non obese (BMI < 25) and obese (BMI > 25) (see Additional file 1: Table S2, S3). Concerning the differences between groups shown, LDL was nearly significant elevated in controlled diabetic group compared to uncontrolled diabetics (P = 0.062). It was significantly elevated in uncontrolled male patients compared to controlled male patients P = 0.048. However, TAG was found to be elevated in uncontrolled female patients P = 0.049.

Pearson’s correlation was applied on Lipocalin-2 and HbA1c as shown in Table 4. Insignificant associations were found between LCN2 and study variables (P > 0.05). HbA1c was found to have significant correlation with duration of diabetes, BMI, waist circumference, HDL and total cholesterol. But no significant correlation was found with LCN2.

**Table 4:** Lipocalin-2 and HbA1c correlations with the study variables

	Lipocalin 2 (ng/dl)		HbA1c	
	Pearson’s correlation	P value	Pearson’s correlation	P value
Duration	0.037	0.786	0.345	0.023
BMI	0.075	0.595	−0.402	0.01
Waist circumference	−0.077	0.625	−0.393	0.026
HbA1c	−0.099	0.521	–	–
Serum creatinine	−0.039	0.773	−0.138	0.372
LDL	−0.222	0.100	0.213	0.169
HDL	−0.127	0.349	0.427	0.004
Triglyceride	0.099	0.469	0.038	0.81
Total cholesterol	−0.104	0.447	0.401	0.008
Age	−0.128	0.341	−0.143	0.354
Lipocalin-2	–	–	−0.099	0.521

## Discussion

This study was designed to find the effect of glycemic control and obesity on lipocalin-2 in patients with type 2 diabetes mellitus.

Obesity, determined by BMI, was insignificantly correlated with LCN2 in this study. This was against to Wang study, which found a strong positive correlation between LCN2 and BMI [24]. Moreover, we found no significant difference in LCN2 between obese and non obese patients with diabetes. This was comparable to El-mesallamy findings [25]. We found that BMI was significantly elevated in female patients with diabetes compared to males in the same group.

Waist circumference was nearly significant difference between patients with diabetes and controls in this study (P = 0.063). However, there were no standard measurements for Africans or Middle Eastern population. The increased waist circumference may increase the liability of patients with diabetes to develop metabolic syndrome as reported earlier [5]. In a study done at the Shanghai diabetic institute, LCN2 was significantly correlated to metabolic syndrome indicated by waist circumference [15]. Another study agreed with the earlier one done by Lee in Seoul, South Korea [26]. In this study, LCN2 was not correlated to waist circumference. Interestingly 88.9% of the women in this study had their waist circumference exceeding 80 cm (according to European standard), although some of them were within the normal weight.

In this study, there was significant elevation HbA1c in patients compared to controls. Moreover, there was significant elevation in HbA1c in diabetic males compared to females of the same group at P = 0.028. This suggested poor glycemic control in males compared with females, which might be due to unregulated diet as they spend most of the day at work. LCN2 was found to be not significantly different between controlled and uncontrolled DM2 which was the same finding reported by El-mesallamy in Egypt [25]. This finding contradicts with the earlier study in China where a strong positive correlation between LCN2 and HbA1c was reported [24]. There was a significant elevation in LDL level in uncontrolled males compared to controlled males in patients with diabetes at P ~ 0.048. That was similar to the result reported by Mohammed in Sudan [27]. However, Mohammed found a nearly significant difference in LDL between controlled and uncontrolled diabetic females, while in this study it was found to be not significant [27]. Instead, we found a significant elevation in total cholesterol in uncontrolled DM2 in female compared to controlled ones P = 0.049.

LCN2 was significantly elevated in patients with diabetes compared with controls. This was the same as the result reported by studies done in Hong Kong and Egypt [24, 25]. There was an insignificant difference in LCN2 between males and females diabetic group and this was different from the finding in China where a significant difference in LCN2 between males and females was found [15, 28]. In this study, the correlation between LCN2, HbA1c and TAG was not significant while it was of strong positive correlation in Wang study [24]. Moreover, there was strong negative correlation between HDL and LCN2 in Wang study, but it was not significant in this study [24]. LCN2 was found as a biomarker of diabetic kidney diseases in urine as reported in studies done in Ohio and Brazil [17, 18]. In this study, no significant correlation between LCN2 and serum creatinine was found.

## Conclusion

Sudanese diabetic patients had elevated levels of serum lipocalin-2. This might indicate their susceptibility to develop complications associated with LCN2 such as metabolic syndrome, insulin resistance, ischemic heart diseases and diabetic kidney diseases. However, there was no significant association found between LCN2 and glycemic control nor obesity. More studies are needed with larger sample size targeting LCN2 association with diabetic complications.

## Additional file

**Additional file 1: Table S1.** Clinical and Laboratory characteristics of DM2 patients and controls according to gender distribution (mean ± standard deviation). **Table S2.** Comparison of clinical and laboratory variables in patients group according to HbA_1c_% (mean ± standard deviation). **Table S3.** Comparison of clinical and laboratory variables in patients group according to BMI (mean ± standard deviation).

## Abbreviations

LCN2: lipocalin-2
NGAL: neutrophil gelatinase-associated lipocalin
DM2: diabetes mellitus type 2
HbA1c: glycated Hemoglobin
BMI: body mass index
LDL: low density lipoprotein
HDL: high density lipoprotein
TAG: triacylglycerol
ELISA: enzyme linked immunosorbent assay
