# Supplementary material for: Association of lipocalin-2 level, glycemic status and obesity in type 2 diabetes mellitus
Source: BMC Res Notes. 2017 Jul 14;10:285. doi: 10.1186/s13104-017-2604-y (PMC5513122; doi:10.1186/s13104-017-2604-y)
Supplement: Supplementary file 1 — Additional file 1: Table S1. Clinical and Laboratory characteristics of DM2 patients and controls according to gender distribution (mean ± standard deviation). Table S2. Comparison of clinical and laboratory variables in patients group according to HbA1c% (mean ± standard deviation). Table S3. Comparison of clinical and laboratory variables in patients group according to BMI (mean ± standard deviation). [file 13104_2017_2604_MOESM1_ESM.docx]

Table S1: Clinical and Laboratory characteristics of DM2 patients and controls according to gender distribution (mean ± standard deviation) .

|  | Patients | | Controls | | ***P1*** | ***P2*** | ***P3*** |
| --- | --- | --- | --- | --- | --- | --- | --- |
|  | Males | Females | Males | Females |  |  |  |
| ***N*** | 26 | 31 | 18 | 12 |  |  |  |
| BMI (kg/m^2^) | 25±4.2 | 30.3±4.9 | 26.4± 4.4 | 28.9±6.5 | <0.001 | 0.328 | 0.461 |
| Waist circumference | 95.2±12.2 | 97.5± 9.5 | 89.7±11 | 93.8±13 | 0.490 | 0.165 | 0.347 |
| HbA1c (%) | 10.2± 2.5 | 8.7± 2 | 5.7±0.9 | 5.8±0.8 | 0.028 | <0.001 | <0.001 |
| Serum creatinine (mg/dl) | 1.1±0.2 | 0.9±0.4 | 0.7±0.2 | 0.8±0.1 | 0.099 | <0.001 | 0.297 |
| LDL (mg/dl) | 87.6±28.2 | 91.2±35.4 | 70.4±26.3 | 59.4±31.5 | 0.587 | 0.048 | 0.008 |
| HDL (mg/dl) | 50.3±17 | 46.6±18 | 62.1±14.1 | 57.8±13.8 | 0.429 | 0.02 | 0.06 |
| Triglyceride (mg/dl) | 140.4±45.5 | 131±58.1 | 153.4±57.6 | 118.9±38.6 | 0.508 | 0.409 | 0.511 |
| Total cholesterol (mg/dl) | 171.5±38.9 | 170.7±41.4 | 158.7±25.5 | 140.9±34.5 | 0.94 | 0.227 | 0.034 |

- P1 for male and female patients, P2 for the male patients and male controls, P3 for the female patients and female controls.

Table S2: Comparison of clinical and laboratory variables in patients group according to HbA_1c_% (mean ± standard deviation)

|  | **Controlled T2DM** | **Uncontrolled T2DM** |  | ***P*** |  |
| --- | --- | --- | --- | --- | --- |
| HbA1c (%) | 6.4 ± 0.54 | 9.9 ± 2 |  | <0.001 |  |
| BMI | 30± 4.5 | 28 ± 5.5 |  | 0.38 | |
| Waist circumference(cm) | 100 ± 4.8 | 98.5 ± 10.6 |  | 0.732 | |
| Serum creatinine (mg/dl) | 1.0± 0.4 | 0.9 ± 0.3 |  | 0.681 | |
| LDL (mg/dl) | 66.8± 33.2 | 88.9 ± 28.6 |  | 0.062 | |
| HDL (mg/dl) | 40.1 ± 14 | 45.9 ± 14.9 |  | 0.322 | |
| Triglyceride (mg/dl) | 128 ± 56.4 | 136.2 ± 55.2 |  | 0.708 | |
| Total cholesterol (mg/dl) | 150.8 ± 45.9 | 166.4 ± 31.3 |  | 0.251 | |

Table S3: Comparison of clinical and laboratory variables in patients group according to BMI (mean ± standard deviation)

|  | ***Non obese***  ***N=17*** | ***Obese***  ***N= 36*** | ***P 1*** |
| --- | --- | --- | --- |
| BMI | 22.5± 1.9 | 30.4± 4.3 | <0.001 |
| Waist circumference | 87.8± 9.1 | 99.8± 9.4 | 0.001 |
| HbA1c (%) | 9.9±2.5 | 9.3± 2.4 | 0.482 |
| Serum creatinine (mg/dl) | 1±0.2 | 0.9±0.4 | 0.661 |
| LDL (mg/dl) | 80.6± 30.8 | 96.2± 31.4 | 0.097 |
| HDL (mg/dl) | 48.9± 17.7 | 48.3±18.4 | 0.912 |
| Triglyceride (mg/dl) | 130.3 ± 48.7 | 140.3± 56.2 | 0.533 |
| Total cholesterol (mg/dl) | 168.6± 38.2 | 171.9± 41.5 | 0.78 |
